# Development, Content Validity and Reliability of Upper Extremity Functional Skill Measure in C5-C7 Spinal Cord Injury

**DOI:** 10.7759/cureus.37599

**Published:** 2023-04-14

**Authors:** Priyanka Vijay, Rajendra Kumar Sureka

**Affiliations:** 1 Department of Occupational Therapy, Mahatma Gandhi Occupational Therapy College, Mahatma Gandhi University of Medical Sciences and Technology, Jaipur, IND; 2 Department of Neurology, Mahatma Gandhi University of Medical Sciences and Technology, Jaipur, IND

**Keywords:** skill measure, rehabilitation, spinal cord injury, activities of daily living, hand functions

## Abstract

Study design: A methodological research design.

Objective: To create an objective measure for assessing hand functions in C5-C7 spinal cord injury (SCI) and estimation of its content validity and internal consistency reliability.

Method: This study was executed in three phases. Phase 1 included a thorough review of the literature, semi-structured in-depth interviews of participants with tetraplegia and interviews of caregivers of SCI individuals and healthcare workers dealing with SCI to understand the hand functions of individuals with C5-C7 SCI. Phase 2 consisted of the development of the tool. The content validity ratio (CVR) method and the opinion of the expert validated the content of the upper extremity functional skill measure (UEFSM). Phase 3 included a quantitative evaluation of the tool which was done on a targeted group of 30 subjects with C5-C7 SCI.

Results: Through the review of the literature and in-depth interview of the participants, 11 items were developed under four content areas: grasp, grip, pinch and gross movement. Items with a minimum CVR of 0.56 were retained at a significance level of p = 0.05 resulting in a 10-item tool for assessing the hand function of individuals with C5-C7 SCI categorized under four subscales. Pilot testing on 10 subjects reveals an average time of 2 minutes and 25 seconds to complete the task. The Cronbach’s alpha was found to be 0.878.

Conclusion: UEFSM is a 10-item tool with good content validity and internal consistency reliability for the assessment of hand functions in individuals with C5-C7 SCI.

## Introduction

Spinal cord injury (SCI) is defined as damage to any part of the spinal cord or the nerves at the end of the spinal canal. It may cause a temporary or permanent alteration in the usual motor, sensory, or autonomic function of the spinal cord [1]. It is a catastrophic event that disrupts every aspect of life including physical and mental [2]. According to estimates, there are around 17,810 new instances of SCI each year in the United States, or 54 cases per million people, excluding those who pass away at the scene of the accident [3]. There is an insufficiency of reliable statistics concerning spinal injuries in India [3-5]. Few research, nevertheless, has provided a summary of the characteristics of SCI individuals [6-9].

The level at which the injury or lesion occurs and the extent (incomplete or complete) of the lesion determine the degree of independence of the individual [10]. Most persons with SCI are thought to have cervical SCI, which makes up about 50% of all cases [11].

The extent and severity of the injury determine the function of the arm and hand after SCI, particularly in tetraplegia [12]. The upper extremity is crucial for several activities of daily living (ADLs) and also contributes to several sports and leisure pursuits that involve multiple joints and both the musculoskeletal and neurological systems. This suggests that upper extremity dysfunction may have a significant effect on autonomy and quality of life, affecting muscle strength, mobility, and coordination [13].

There are several generic hand function standardized instruments like Jebsons Taylor Hand Function Test (JHFT), Perdue pegboard test, etc., that are being used for the assessment of hand functions. Among these, the JHFT is a commonly used hand function evaluation instrument in the majority of clinical contexts. Tetraplegics' activities and hand grips vary from those of other patient populations. Researchers [14-16] advised against using these tests in SCI clinical practice and research investigations because there are no guidelines for scoring if the participant dropped the item(s), used a different grasp pattern, or took longer than the allotted time to complete the task.

Another hand function measure, Sollerman Hand Function Test [17], assesses 20 daily living activities that require the most typical gripping actions. According to previous studies [14], individuals with C5 to C7 SCI are frequently unable to execute the required seven grasp methods, which causes them to underperform with this measurement tool, making the Sollerman Hand Function Test manual an inappropriate test for assessing hand function in these individuals.

The Capabilities of Upper Extremity Questionnaire (CUE-Q) [18] is a 17-item questionnaire in which individual evaluate their ability to use the arms for functional tasks on a 7-point ordinal scale. The CUE-Q effectively assesses outcomes following surgery for enhancing upper limb functions by differentiating between the proximal arm and hand function. The Motor Capacity Scale (MCS) [19], regardless of context or setting, the test assesses motor skills that are comparable to the functional tasks that people with SCI usually perform. These measures focus mainly on carrying out activities that demand stability and bodily movements beyond hand grasp and release.

Another disease-specific assessment tool that assesses upper limb functions in SCI is the Tetraplegia Hand Activity Questionnaire (THAQ) [20]. The Delphi Method [21] was used to construct the THAQ, which led to the incorporation of arm-hand tasks that are significant to SCI measuring activities related to daily living tasks. The THAQ has not been reported to have any clinical applications.

The Grasp and Release Test (GRT) was developed for measuring hand function in individuals with tetraplegia. The GRT evaluates three characteristics: pinch force, grab force, and hand function. The psychometric properties of the test were established [14,22]. Intra-class correlation coefficients for repeated GRT measures were high [22]. GRT scores were predictable over time and responsive to alterations in hand function through the use of FES and tendon transfers for chronic stable hand-function assessment [22-25]. Since the primary goal of the GRT was to assess changes produced by FES palmar and lateral grasp, it may be insensitive to alternative grasp patterns and/or levels of injury that do not usually employ current FES systems (such as high and low cervical SCI), and some of the lighter objects (peg and block) show no difference before and after surgery. Additionally, the GRT eliminates proximal contributions to hand functions.

Graded Redefined Assessment of Strength, Sensation, and Prehension (GRASSP) [26], another performance-based outcome measure designed especially for SCI individuals and found to be accurate for evaluating hand function in the SCI population. However, in the C5 SCI population, the GRASSP is not well adapted for evaluating the force exerted during a unilateral lateral pinch, pulp pinch, and palmar grasp. The assessment of prehension in GRASSP is subjective. The expense of the GRASSP kit and the lengthened completion time result in a reduction in the clinical utility of this measure [27].

Individuals with C5-C7 SCI often have specific patterns of hand function impairment, which may not be captured by more general hand function assessment tools. Hand assessments designed for other populations may not take into account the unique challenges faced by individuals with SCI, such as muscle weakness, spasticity, and reduced range of motion. There are a number of evaluation tools for tetraplegics that measure hand functions, however, the literature search, to the best of my ability, found that the majority of them assess hand functions indirectly and primarily by focusing on how well the subject performs daily activities. These scales may not capture the full range of functional abilities and limitations experienced by individuals with SCI, which can vary widely depending on the level and severity of the injury. It is important to choose an evaluation tool that is appropriate for the individual's level of injury, functional abilities, and goals of rehabilitation. The feasibility of the tool, including the time required to administer and cost, should also be considered. Hence, the objective of this study was to develop a comprehensive measure for the assessment of hand functions in individuals with C5-C7 SCI.

## Materials and methods

The study was carried out in three phases [28].

PHASE 1: Planning

PHASE 2: Construction

PHASE 3: Quantitative Evaluation

**Figure 1:**
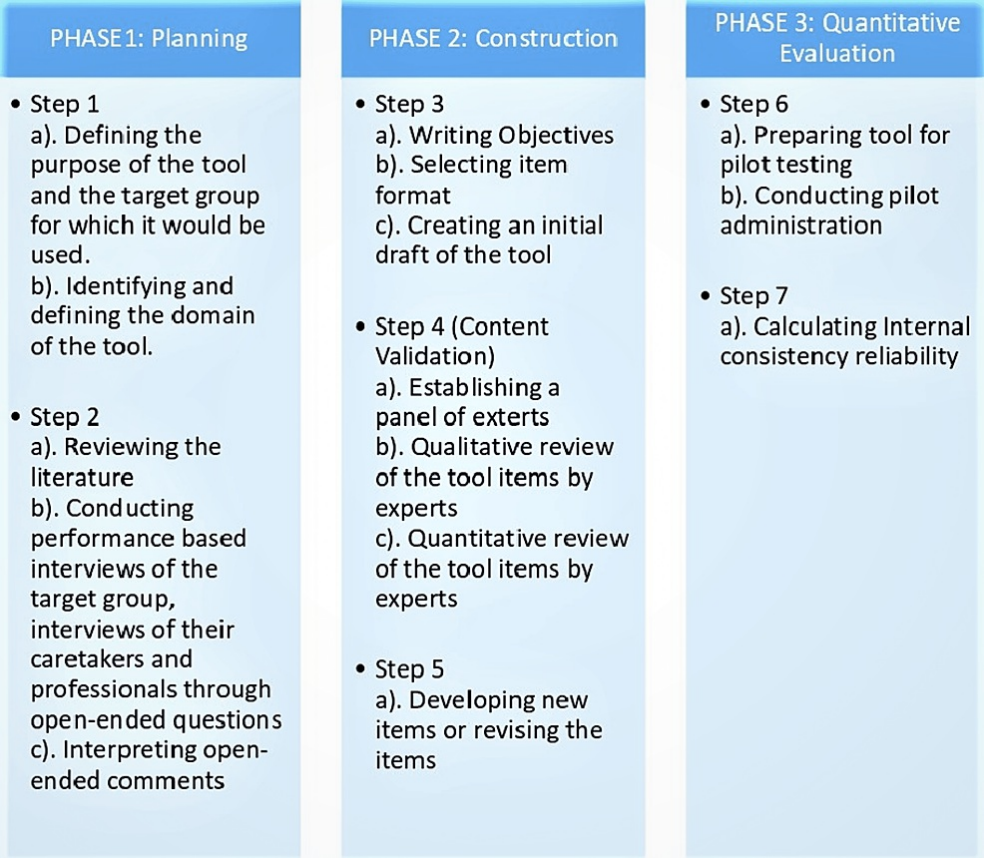
Flowchart of the phases of the study

The phases of the study can be seen pictorially in the form of a flowchart in Figure 1.

Planning phase (steps 1 and 2)

The first step began by defining the purpose of the tool, which was specified as to evaluate the hand functions of individuals with C5-C7 SCI. Hand function was the domain to be measured, and the target group was identified as individuals with C5-C7 SCI.

The second step involved a review of related literature in order to understand the various aspects of hand functions such as sensory-motor components involved in the hand functions, the functional task performed by hand, the issues related to upper extremity impairment in individuals with C5-C7 SCI, and the scales/tools that already exist for the assessment of hand functions. The process of the literature review was accomplished through PubMed, PMC, SCOPUS and Web of Science.

After the identification of the types of items to assess the hand function, open-ended questions were prepared for one-to-one interviews with individuals with C5-C7 SCI, their caretakers and health professionals dealing with SCI clients to gain thorough knowledge regarding the domain of hand functions. During the semi-structured interview, the respondents were asked open-ended questions regarding the hand functions of the individual with C5-C7 SCI. On asking about the activities/circumstances that challenge or disturb the hand functions of individuals with C5-C7 SCI, more questions were added and rephrased regarding specific activities of daily living performed with hands. The subjects were also asked to perform certain movements of the upper extremities like raise your arm, bend your elbow and make a grasp, etc. so that the information obtained from the interview and movements performed could help in generating a pool of appropriate items.

Construction phase (steps 3-5)

Third step - Based on the literature review and in-depth interviews, the content areas (grasp, grip, pinch, and gross movement) of the tool were thus identified and items of each area were generated. Four content areas were developed through the overall process. ‘Grip’ contained two items and ‘grasp’, ‘pinch’, and ‘gross movement’ contained three items each. Scoring was formed using the ordinal scale for obtaining the individual scores of the items which could be added to determine the total score of the measure. The score of each item ranged from 0 to 4.

The fourth step involved evaluating content validity which was done using the qualitative and quantitative review of the tool by the expert panel. In the qualitative review, the experts were requested for their opinions on the title of the tool, instructions, domain considered, item scoring, and the overall items of the tool. In the quantitative review, experts were requested to rate each item of the tool on the essentiality of the items. Using the Lawshe formula, the content validity ratio (CVR) was determined for each item [29].

After implementing the changes suggested by experts and comparing the calculated CVR to the values required for statistical significance at p = 0.05, the final form of the tool was prepared for quantitative evaluation.

Quantitative evaluation phase (steps 6 and 7)

The tool was then administered to a group of individuals with C5-C7 SCI. Medically stable subjects aged 18 years and above with C5-C7 SCI (ISNCSCI A-D) were included in the study. Subjects who were unable to understand the command, any diagnosed psychiatric illness, and any other diagnosed condition which affects hand functions were excluded. Ten subjects were included for pilot testing and the time taken to perform each item was recorded. Then 20 individuals with C5-C7 SCI were recruited using purposive sampling for item analysis.

## Results

Phase 1

The respondents generally mentioned performing hand functions with tenodesis grasp. They have also mentioned that the pinch is more effective with lateral prehension as compared to a tripod or tip-to-tip and gross movements are easier in diagonal patterns as compared to movement occur in plane and axis.

Phase 2

Based on the review of the literature and in-depth interview, the hand function tool was constructed under four content areas.

Content validity of ‘upper extremity functional skill measure’ (UEFSM).

The initial draft of the tool consisted of 11 items in total. Given that there were 12 experts, a CVR of 0.56 was required as a minimum for satisfying the 5% level [29]. CVR of the items of the initial form of the tool is shown in Table 1.

**Table 1:** Content validity ratio for items of the initial form of the tool

S. no.	Content area	Item number	Content validity ratio (CVR)
1	Grasp	1	0.83
		2	0.66
		3	1
2	Grip	4	1
		5	0.33*
3	Pinch	6	1
		7	1
		8	1
4	Gross movement	9	0.66
		10	0.83
		11	1

Out of 11 items, the CVR values of 10 items were greater than 0.56 (shown in Table 1) indicating that these items were proved to have content validity. Item number five had a CVR value of 0.33* which is below 0.56, so, this item was dropped out. This item was related to carrying a weight of 200 gm. The experts have rated this item ‘not essential’ as they commented that instead of representing functional activities of the hand, this item is more useful to measure strength. Thus, the process of calculating the CVR of individual items of the tool resulted in the 10-item tool (Table 2).

**Table 2:** Content validity ratio (CVR) for items of the final form of the tool

S. no.	Content area	Item number	CVR
1	Grasp	1	0.83
		2	0.83
2	Grip	3	1
		4	1
3	Pinch	5	1
		6	1
		7	1
4	Gross movement	8	0.83
		9	0.83
		10	1

Phase 3

A total of 10 individuals with C5-C7 SCI (Table 3) were purposively sampled and pilot testing of the scale was conducted and the time taken to perform each item was noted (Table 4).

**Table 3:** Demographic characteristics of participants (Pilot test)

Demographic characteristics		N
Age (in years) (Mean ± SD = 38.20 ± 22.77)	18-38	7
	39-59	0
	60-80	3
Gender	Male	9
	Female	1
AIS Grade (ISNCSCI)	A	5
	B	1
	C	3
	D	1
Mode of injury	Traumatic	7
	Non-Traumatic	3
Duration of injury (in months)	0-12	6
	13-24	2
	25 and above	2
Duration of rehab (in months)	0-12	7
	13-24	1
	25 and above	2

**Table 4:** Descriptive statistics (mean of time and standard deviation of items for N=10)

Item no. and description	Mean	S.D.	Min.	Max.	Range	Confidence interval at 95%	
						Lower bound	Upper bound
1. Stacking four glasses that accommodate at least 3 of the subject’s fingers	0.17	0.08	0.10	0.29	0.19	0.18	0.23
2. Placing ball on the shelf of the kit	0.06	0.04	0.02	0.12	0.10	0.03	0.09
3. Arranging four alloy tubes of difference size in ascending order	0.22	0.09	0.13	0.36	0.23	0.15	0.29
4. Stacking different size of wooden blocks	0.11	0.08	0.03	0.23	0.20	0.05	0.18
5. Transfer four beads from one tray to the another tray	0.13	0.07	0.06	0.23	0.17	0.08	0.18
6. Writing name	0.27	0.10	0.12	0.42	0.30	0.19	0.34
7. Opening bottle cap	0.15	0.08	0.06	0.26	0.20	0.09	0.21
8. Placing hand overhead	0.04	0.01	0.02	0.05	0.03	0.03	0.04
9. Placing hand on top shelf (at shoulder height) of the kit	0.02	0.00	0.01	0.03	0.02	0.02	0.02
10. Hand to mouth	0.02	0.00	0.01	0.02	0.01	0.02	0.02

Then 20 subjects with C5-C7 SCI (Table 5) were recruited with purposive sampling for internal consistency reliability. The Cronbach’s alpha for 10 items was found to be 0.878. The inter-item correlations ranged from 0.035 to 0.856 at a 5% significance level. All items showed a positive correlation with each other (Table 6).

**Table 5:** Demographic characteristics of participants

Demographic characteristics		N
Age (in years) (Mean ± SD = 32.35 ± 9.92)	18-30	10
	31-43	7
	44-56	2
	57-69	1
Gender	Male	18
	Female	2
AIS Grade (ISNCSCI)	A	12
	B	6
	C	2
	D	0
Mode of injury	Traumatic	19
	Non-Traumatic	1
Duration of injury (in months)	0-12	7
	13-24	3
	25 and above	10
Duration of rehab (in months)	0-12	12
	13-24	2
	25 and above	6

**Table 6:** Inter-item correlation of 10 items

	Item 1	Item 2	Item 3	Item 4	Item 5	Item 6	Item 7	Item 8	Item 9	Item 10
Item 1	1									
Item 2	.476	1								
Item 3	.341	.196	1							
Item 4	.773	.035	.551	1						
Item 5	.448	.649	.603	.324	1					
Item 6	.557	.742	.660	.300	.636	1				
Item 7	.615	.481	.604	.456	.680	.669	1			
Item 8	.476	.758	.512	.316	.856	.700	.547	1		
Item 9	.143	.631	.512	.063	.701	.700	.547	.818	1	
Item 10	.212	.094	.475	.094	.346	.283	.393	.405	.270	1

Hence, the study resulted in the 'Upper Extremity Functional Skill Measure', a performance-based tool with 10 items being scored by the investigator on an ordinal range from 0 to 4. A score of '0' depicts no hand movements, while a score of '4' implies normal movement. The average time taken to administer the UEFSM during the pilot test was 2 minutes and 25 seconds. A table, a chair or wheelchair, and a tool kit consisting of glasses, a small ball, alloy tubes, different-sized wooden blocks, a pen, beads, and a small bottle are needed for the administering of UEFSM.

## Discussion

Hand function is one of the most important components in maintaining independence in daily life. The assessment of hand function is crucial in cervical SCI, as it provides important information concerning the extent of damage and the potential for recovery. There is no single standardized test that comprehensively measures all of the many hand functions of individuals with C5-C7 SCI who are partially or completely unable to use their hands in various activities. Hence, the objective of this study was to develop a tool for assessing the hand functions of an individual with C5-C7 SCI and determine its reliability and validity.

This study resulted in the development of the UEFSM designed to assess the hand functions of individuals with C5-C7 SCI. The UEFSM consisted of four subscales namely, grasp, grip, pinch, and gross movement. The items included in these subscales assess an individual's ability to manipulate objects that they might regularly come into contact with. Detailed description and rationale of the item consisting in UEFSM is given in Table 7.

**Table 7:** Detailed description and rationale of the item in UEFSM *Subscale IV requires no specialized item as it requires to perform the simple gross movement. UEFSM: Upper Extremity Functional Skill Measure; ADL: activities of daily living

Subscale	Item no. and object	Description	Rationale
I. Grasp	1. Four Glasses (200 ml capacity each)	Stacking glasses that accommodate atleast 3 of the subject’s finger (representing cylindrical grasp)	Mimics holding a glass or arranging something in order - a typical ADL task
	2. Ball (7.5 diameters)	Placing ball on the shelf of the kit (representing spherical grasp)	Mimics holding a bowl - a typical ADL task
II. Grip	3. Four different size alloy tubes (length - 5cm, 6cm, 7cm and 8 cm)	Arranging alloy tubes in ascending order (representing lateral prehension grip using eye-hand coordination and perception)	Mimics holding a spoon
	4. Three different size wooden blocks (2 inches, 1.5 inches and 1 inch)	Stacking wooden blocks (representing lumbrical grasp)	Mimics holding a book
III. Pinch	5. Four beads (2cm in diameter)	Transfer beads from one tray to the another tray (representing pulp pinch)	Mimics picking up with the help of finger and placing it on the other side crossing midline
	6. A writing pen	Writing name (using tripod pinch)	A typical ADL task
	7. A small bottle (50ml capacity)	Opening bottle cap (in-hand manipulation, rotation component)	Mimics opening the hair oil bottle which is a typical ADL task
IV. Gross Movement*	8.	Placing hand overhead	Mimics reaching or taking out something from upper shelf
	9.	Placing hand on top shelf (shoulder height) of the kit	Mimics reaching or taking out something with forward reach
	10.	Hand to mouth	Simulated eating

While administering the test, place each test item in front of the participant on a table. The object must be grasped by the participant, lift completely off from the supporting surface, manipulate it, and then place back on the table or shelf of the kit.

The 5-point ordinal scoring system is based on an objective evaluation of performance. Reach, grip, and manipulation are the three key elements of grasp and manipulation that are taken into consideration in the scoring system. This scoring system is applied to Items 1 to 10. The scoring system for UEFSM (scale 0-4) is shown in Table 8.

**Table 8:** Scoring of the tool

Score	Description
0	Cannot perform any part of the test
1	Perform the test partially with great difficulty
2	Perform the test partially with ease
3	Complete the test, but takes abnormally longer time
4	Perform the test normally

An occupational therapist or physiotherapist who specializes in the hand or upper extremities should preferably administer the UEFSM. The administration time of this 10-item measure is yet to be determined. The 10-item UEFSM, which was used in the pilot study, took an average of 2 minutes and 25 seconds to complete.

UEFSM is simple to use and requires fewer readily available tools that can be found in hardware and retail stores. Tools and materials that are readily available can be used to create those items that require minimal carpentry. These equipments used in UEFSM are cost-effective as against GRASSP and GRT. These assessment tools are not frequently used in clinical or rehabilitation settings because they are expensive, require specialized equipment, and require training for the clinician using them to gather and analyze data. Because GRASSP and GRT are not available in all clinical set-ups, therapists evaluating hand function typically use a subjective score of normal, good, fair, or poor. However, the validity and reliability of this method have not been established.

UEFSM is an objective measure of hand functions in individuals with C5-C7 SCI as it provides an ordinal score for all items which needs to be performed. The score of the individual items is added to obtain the total score of the overall measure, indicating the hand function performance of the C5-C7 SCI individual.

UEFSM will objectively assess the remaining hand functions of a person with C5-C7 SCI, which was the main objective of the study. The tool does not evaluate the underlying neuromuscular physiological changes [30] post-SCI leading to hand dysfunction. The content validity of the UEFSM has been established (CVI=0.93) and the Cronbach alpha was found to be 0.878 which shows good internal consistency reliability. UEFSM provides a clear picture of the hand function components in which the performance of the individual with C5-C7 SCI is impaired.

Limitation and recommendation

The sample size was not statistically estimated for the pilot test due to the lack of a reliable database of SCI in India. Future studies on the estimation of further psychometric properties of ‘Upper Extremity Functional Skill Measure’ are recommended. Future studies can be done on the development of outcome measures of hand functions in SCI in acute settings.

## Conclusions

The findings of the study suggest that the 'Upper Extremity Functional Skill Measure (UEFSM)' is a simple and easy-to-administer tool that can provide an objective measure of hand function in individuals with C5-C7 SCI. The ease of administration and simplicity of the tool make it more feasible for use in clinical settings and can save time and resources. This can be particularly important in settings with limited resources, where efficiency and effectiveness are critical for providing optimal care. The content validity of UEFSM is established and item analysis shows a positive inter-item correlation. Further psychometrics need to be established before it is used in clinical settings.
